# The regulatory roles of long non-coding RNAs in the development of chemoresistance in breast cancer

**DOI:** 10.18632/oncotarget.22577

**Published:** 2017-11-08

**Authors:** Akshay Malhotra, Manju Jain, Hridayesh Prakash, Karen M. Vasquez, Aklank Jain

**Affiliations:** ^1^ Center for Biochemistry and Microbial Sciences, Central University of Punjab, Bathinda, Punjab, India; ^2^ Laboratory of Translational Medicine, School of Life Sciences, University of Hyderabad, Gachibowli, Hyderabad, India; ^3^ Division of Pharmacology and Toxicology, College of Pharmacy, The University of Texas at Austin, Dell Pediatric Research Institute, TX, USA; ^4^ Center for Animal Sciences, Central University of Punjab, Bathinda, Punjab, India

**Keywords:** breast cancer, chemoresistance, long non-coding RNA, lncRNA, drug resistance

## Abstract

Chemoresistance is one of the major hurdles in the treatment of breast cancer, which limits the effect of both targeted and conventional therapies in clinical settings. Therefore, understanding the mechanisms underpinning resistance is paramount for developing strategies to circumvent resistance in breast cancer patients. Several published reports have indicated that lncRNAs play a dynamic role in the regulation of both intrinsic and acquired chemoresistance through a variety of mechanisms that endow cells with a drug-resistant phenotype. Although a number of lncRNAs have been implicated in chemoresistance of breast cancer, their mechanistic roles have not been systematically reviewed. Thus, here we present a detailed review on the latest research findings and discoveries on the mechanisms of acquisition of chemoresistance in breast cancer related to lncRNAs, and how lncRNAs take part in various cancer signalling pathways involved in breast cancer cells. Knowledge obtained from this review could assist in the development of new strategies to avoid or reverse drug resistance in breast cancer chemotherapy.

## INTRODUCTION

Despite advances in early diagnosis, therapy and surgery, breast cancer is still the leading cause of cancer related death in women worldwide [1, 2]. Although various chemotherapeutic agents such as docetaxel, tamoxifen, cisplatin, carboplatin, doxorubicin, gemcitabine, mitoxantrone etc. have improved the overall survival and quality of life for patients, the 5 year survival of stage IV breast cancer patients is still very low (∼20%) for most estrogen receptor (ER) positive/progesterone receptor (PR) negative subtypes [3]. This may be in part due to increases in intrinsic (e.g. stromal fibrosis and interactions between cell surface integrins and extracellular matrix components) and extrinsic factors (e.g. the expression of one or more energy-dependent transporters which eject chemotherapeutic drugs from cells) that promote resistance to chemotherapy in breast cancer cells [4]. Thus, there is an urgent need to better understand the mechanisms associated with chemoresistance in breast cancer to aid the development of improved therapies for the treatment of breast cancer.

The advancement in large-scale whole genome sequencing technologies suggests that less than 2% of the human genome encodes for proteins, whereas much of the remaining genome is transcribed into non-coding RNAs (ncRNAs) [5, 6]. Such non-coding RNAs are transcribed into a large variety of regulatory RNAs, including piwi-interacting RNAs (piRNAs), microRNAs (miRNAs), small-interfering RNAs (siRNAs), circular RNAs (circRNAs), small Cajal body-specific RNAs (scaRNAs), small nucleolar RNAs (snoRNAs), transfer RNAs (tRNAs), ribosomal RNAs (rRNAs) and long non-coding RNAs (lncRNAs) [7, 8]. Among these ncRNAs, lncRNAs (i.e. lengths > 200 nucleotides) are found to be important regulators of carcinogenesis [9]. Previous studies have provided evidence for their regulatory roles in facilitating carcinogenesis, invasion-metastasis, and chemoresistance in many cancers, as reviewed in [10]. Their roles in the pathogenesis of gallbladder [11] and lung cancers [5] have also been highlighted in recent reviews, along with their regulatory roles in a wide range of biological processes such as transcription, translation, RNA interference, epigenetic gene regulation, and cell-cycle control.

**Figure 1:**
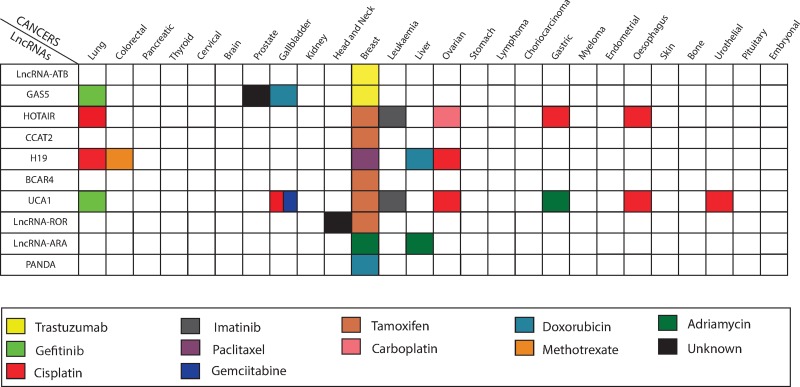
Altered expression level of lncRNAs dysregulates chemotherapy treatment in various human cancers The shaded squares represent a relationship between lncRNAs (on the left) varied chemotherapy response in different cancer types (on the top) in the Figure.

Recently, lncRNAs such as *lncRNA-ATB* [12], *GAS5* [13, 14], *HOTAIR* [15], *CCAT2* [16], *H19* [17], *BCAR4* [18], *UCA1* [19–21], *LncRNA-ROR* [22] and *LncRNA-ARA* [23] have been implicated in resistance to chemotherapy in breast cancer patients (Table 1 and Figure 1). Though these lncRNAs have been implicated in resistance to chemotherapeutic agents used in the treatment of breast cancer, a comprehensive review is still missing. Thus, in this review, we discussed in detail the regulatory roles of various lncRNAs in contributing to chemoresistance in breast cancer therapy. The published work described has provided a better understanding of the mechanisms underlying the contribution of lncRNAs to chemoresistance, which may lay the foundation for the development of improved therapies for the treatment of breast cancer.

**Table 1:** Breast cancer-associated long non-coding RNAs, their reported biological functions in drug resistance and the affected pathways

S.No.	LncRNA	Genomic Location	Mean fold change in expression compared to controls	Resistance against	Property	Validation Methods	Biological significance	Genes/Proteins/ Pathways affected	Cell lines	References
1)	LncRNA-ATB	14q11.2	∼↑2.0 fold	Trastuzumab	Oncogenic	Microarray analysis; qRT-PCR	Promotes cell proliferation, EMT, invasion and metastasis	↑miR200c; ↑Vimentin; ↓E-cadherin; ↑ZEB1; ↑ZNF217; ↑ETS1; ↑TWIST1; ↑SNAIL1; ↑TGF-β ↑TGF-β signaling	SKBR3	[12]
2)	GAS5	1q25.1	∼↓2.7 fold	Trastuzumab	Tumor suppressor	Microarray analysis; qPCR; Western blot analysis	Inhibits cell proliferation and tumor growth	↓PTEN; ↓p53; ↓p27 ↑ miR-21; ↑Akt; ↑Bad; ↑caspase 9; ↑PDK1; ↑PDK2; ↑MDM2; ↑IKK; ↑GSK-3β; ↑mTOR ↑Akt/PI3K/mTOR signaling pathway	SKBR3	[13, 14]
3)	HOTAIR	12q13.13	∼↑4.0 fold	Tamoxifen	Oncogenic	Microarray analysis; qPCR; Western blot analysis	Promotes cell proliferation, invasion and metastasis	↑ EZH2; ↑estrogen receptor protein; ↑ GREB1 gene; ↑TFF1 gene; and↑c-MYC gene	MCF-7 and T47D	[15]
4)	CCAT2	8q24.21	∼↑6.5 fold	Tamoxifen	Oncogenic	Microarray analysis; qRT-PCR; Western blot analysis	Promotes cell proliferation; tumorigenesis and inhibits apoptosis	↑ERK; ↑ ERK/MAPK signaling pathway	MCF-7 and T47D	[16]
5)	H19	11p15.5	∼↑3.0 fold	Paclitaxel	Oncogenic	qRT-PCR; Western blot analysis; ChIP assay	Promotes tumorigenesis, metastasis and inhibits apoptosis	↓BIK gene; ↑EZH2; ↓NOVA gene	MCF-7 and ZR-75-1	[17]
6)	BCAR4	16p13.13	Not determined	Tamoxifen	Oncogenic	qRT-PCR; Western blot analysis	Promotes cell proliferation, metastasis and tumor aggressiveness	↑ERBB2; ↑ERBB3; ↑Akt; ↑ERK1/2; ↑AIB-1; ↓Pax2; ↑ERBB2/ERBB3 signaling pathway	ZR-75-1	[18]
7)	UCA1	19q13.12	∼↑20.0 fold	Tamoxifen	Oncogenic	qRT-PCR; Western blot analysis	Promotes cell proliferation, tumorigenesis and Inhibits apoptosis	↓miR-143; ↓caspase 3; ↓miR-18a; ↓p27; ↑Akt; ↑mTOR; ↑Akt/mTOR signaling pathway; ↑wnt/β-catenin pathway	MCF-7; LCC2; LCC9; BT474; T47D	[19, 20, 21]
8)	LncRNA-ROR	18q21.31	∼↑7.0 fold	Tamoxifen	Oncogenic	qRT-PCR; Western blot analysis	Promotes cell proliferation and invasion	↑ZEB1; ↑ZEB2; ↑vimentin; ↓miR-205-5p; ↓E-cadherin	TR5; MCF-7; MDA-MB-231	[22]
9)	LncRNA-ARA	Xq23	∼↑4.0 fold	Adriamycin	Oncogenic	qRT-PCR; Microarray analysis	Promotes cell proliferation; migration and inhibits apoptosis and cell cycle arrest	↑Cyclin B1; ↑Bcl-xL protein; ↓Bax protein; ↑MAPK signaling; ↑PPAR	MCF-7	[23]

### Long non-coding RNA activated by TGF-β (LncRNA-ATB)

As the name suggests, TGF-β regulates and activates the expression of *LncRNA-ATB*. TGF-β, a master regulator of EMT (epithelial–mesenchymal transition), alters gene expression of several oncogenes such as Zinc finger E-box-binding homeobox 1 (*ZEB1*), Zinc finger E-box-binding homeobox 2 (*ZEB2*) and Zinc finger 217 (*ZNF217*) that promote cellular proliferation, invasiveness, and resistance to apoptosis. *Lnc-ATB* is a non-adenylated 2 exon transcript, which stretches over 80 kb at chromosome 14q11.2 [24]. It has been shown to be significantly over-expressed in several human cancers such as hepatocellular carcinoma [25], colorectal [26, 27], prostate [28], and gastric cancers [29].

**Figure 2:**
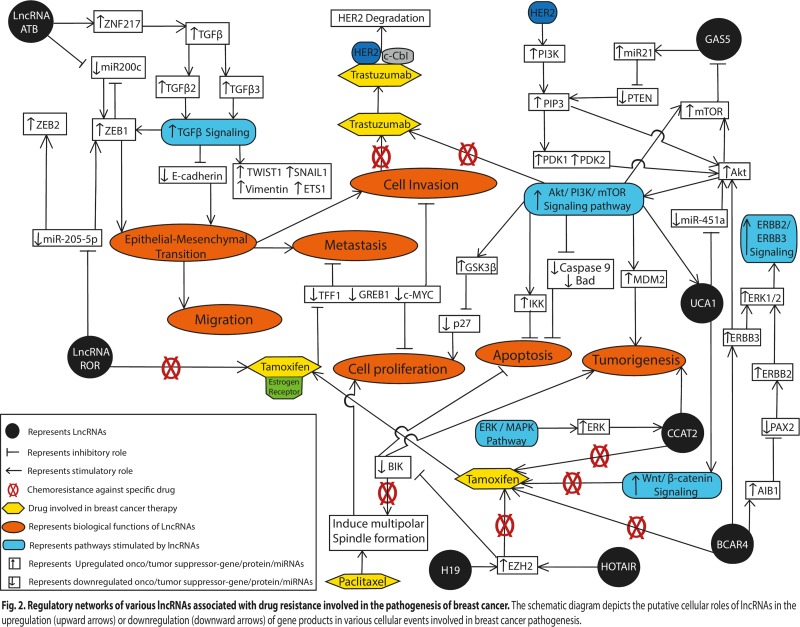
Regulatory networks of various lncRNAs associated with drug resistance involved in the pathogenesis of breast cancer The schematic diagram depicts the putative cellular roles of lncRNAs in the upregulation (upward arrows) or downregulation (downward arrows) of gene products in various cellular events involved in breast cancer pathogenesis.

Approximately 25% of patients with metastatic HER2-amplified breast cancer, which is the leading cause of mortality in breast cancer patients, show resistance to trastuzumab based therapy [12, 30]. Shi et al. (2015) found that *lncRNA-ATB* was up-regulated by ∼2-fold in breast cancer patients on trastuzumab therapy over other breast cancer patients, by using microarray analysis on breast cancer tissues of five trastuzumab-resistant (TR) breast cancer patients [12]. Similarly, they observed that TR human SKBR-3 breast cancer cells (TR SKBR-3) expressed 2.5-fold more *lncRNA-ATB* compared to wild-type SKBR-3 (WT SKBR-3) breast cancer cells. It was further observed by the authors that in the presence of trastuzumab, TR SKBR-3 cells had a greater rate of migration and invasion compared to WT SKBR-3 cells, and showed lower apoptotic rates in the presence of trastuzumab [12]. Moreover, down-regulation of *lncRNA-ATB* by shRNA-ATB significantly decreased the migration and invasion capacity of the TR SKBR-3 cells, which suggested that *lnc-ATB* promoted trastuzumab resistance by facilitating the invasive and EMT characteristics of these breast cancer cells. Earlier reports suggested that *lncRNA-ATB* functions as a competing endogenous RNA (ceRNA) and competes with miR-200c binding sites. miR-200c has been reported to repress EMT and tumor invasion by targeting the 3′UTR of the ZEB1 and ZNF217 genes [31] (Figure 2). The authors determined the relative expression levels of miR-200c and *lncRNA-ATB* in breast cancer cells by performing microarray experiments and found that the expression level of miR-200c was significantly downregulated in both TR breast cancer patients (∼2-fold) and TR SKBR-3 cells (2.5-fold) compared to their respective control, while *lnc-ATB* levels were increased. Further, by luciferase assays, the authors demonstrated that ectopic expression of lncRNA-ATB in TR SKBR-3 cells decreased miR-200c expression; whereas, silencing of lncRNA-ATB increased miR-200c expression. This result implied that *lncRNA-ATB* binds to miR-200c and inversely correlates with its expression in TR SKBR-3 cells. *LncRNA-ATB* shares a regulatory miR-200c sequence within ZEB1 and ZNF217, and it was found that up-regulation of *lncRNA-ATB* led to an increase in the expression of ZNF217 and ZEB1 at both the mRNA and protein levels in TR SKBR-3 cells [12]. It was further observed that the up-regulation of ZEB1 and ZNF217 by lnc-ATB further augmented the EMT effects in breast cancer cells (Figure 2). ZEB1, a key mediator of TGF-β signaling and EMT regulatory pathways, directly suppresses miR-200c by targeting at least two highly conserved ZEB1-binding sequences that are located within the putative promoter; hence over-expression of ZEB1 leads to inhibition of miR-200c promoter activity (Figure 2). Moreover, enhanced TGF-β signaling up-regulates ZEB1 expression levels, and ZNF217 acts as an enhancer of TGF-β, and transcriptionally activates TGF-β2 and TGF-β3 in an autocrine loop [9, 32] (Figure 2).

Taken together, the findings above suggest that a high level of *lncRNA-ATB* correlated with trastuzumab resistance in breast cancer patients, and it has pleiotropic effects on breast cancer cell invasion and trastuzumab resistance. Thus, it might be considered as an effective target molecule for anti-metastasis and reversal of trastuzumab resistance therapies for breast cancer patients.

### Growth arrest specific transcript (GAS5)

*GAS5* is a non-protein coding gene, which spans ∼7.0 kb on chromosome 1q25.1 and comprises a large number of small nucleolar RNAs (snoRNAs) [33]. It contains 10 C/D box snoRNAs, which are associated with methylation as well as 12 weakly conserved exons that are located within highly conserved introns, along with a 5′-terminal oligopyrimidine tract [33]. In humans, the *GAS5* gene is expressed ubiquitously in many cell types and plays an important role in tumorigenesis [34]. Several studies have demonstrated that *GAS5* is down-regulated in gastric [35], glioma [36], stomach [37], lung [38], cervical [39], liver [40], gallbladder [41], pancreatic [42], prostate [43] and breast cancer [13]. In breast cancer, *GAS5* expression was found to be significantly down-regulated (∼2.7-fold) in cancerous tissues in breast relative to surrounding non-cancerous tissue, and this reduced expression of *GAS5* was correlated with advanced Tumor-Node-Metastasis (TNM) stage and lower rates of survival in these patients [13]. Moreover, down-regulation of *GAS5* expression is also associated with chemoresistance in lung (gefitinib) [38], gall bladder (doxorubicin) [44] breast (trastuzumab) [13] and in prostate cancers [45] (Figure 1).

Recently, Li et al. (2016) observed that *GAS5* expression was significantly down-regulated (∼2.7-fold) in trastuzumab-resistant TR SKBR-3 cells and trastuzumab-resistant breast cancer patients (*n* = 86) compared to non-tumor tissues, as assessed by real-time PCR [13]. Furthermore, they found that *GAS5* expression was also down-regulated (∼2.0-fold) in HER-2 positive breast cancer samples (*n* = 20) relative to matched noncancerous tissue samples [10]. Toward a better understanding of the mechanistic involvement of *GAS5* in trastuzumab-resistant breast cancer cells, they found that knockdown of *GAS5* led to cell proliferation and tumor growth in SKBR-3 trastuzumab-resistant cancer cells. Knockdown of *GAS5* in trastuzumab-resistant cells also led to decreased phosphatase and tensin homolog (PTEN) expression. PTEN is ubiquitously expressed in humans, where it dephosphorylates phosphatidylinositol-3,4,5-triphosphate (PIP3) and negatively regulates the AKT/PI3K pathway; a signaling pathway known to increase drug sensitivity, including sensitivity of trastuzumab (Figure 2). Further, it was found that *GAS5* functions as a molecular sponge for miR-21, which is known to promote tumor proliferation and invasion by down-regulating PTEN expression (Figure 2). These findings suggest that the down-regulation of *GAS5* can lead to de-repression of the PTEN gene in trastuzumab-resistant breast cancer cells [10]. Thus, *GAS5* may be explored as a prognostic and diagnostic molecule in trastuzumab-resistant breast cancer patients.

### HOX transcript antisense RNA (HOTAIR)

*HOTAIR*, a 2.2 kb transcript localized on the antisense strand of the Homeobox C (HOXC) locus on chromosome 12q.13.13, has been identified as a cancer-associated lncRNA [5]. It interacts with the Polycomb Repressive Complex 2 (PRC2), which induces histone H3 lysine-27 trimethylation at the Homeobox D (HOD) domain, which down-regulates the expression of a number of genes that can result in tumorigenesis and metastasis [46]. This lncRNA was found to be up-regulated in various cancer types, such as ovarian [47], gastric [48], prostate [49], kidney [50], cervical [51], ovarian [52], bladder [53], lung [54] and breast [46]. Over-expression of HOTAIR can result in chemoresistance against certain drugs in ovarian (carboplastin) [47, 55], gastric (cisplatin) [56], lung (cisplatin) [57, 58], leukemia (imatinib) [59], esophagus (cisplatin) [60], and breast (tamoxifen) cancers [15] (Figure 1).

Tamoxifen is among the most commonly used chemotherapeutic agents in the treatment of breast cancer, specifically estrogen receptor (ER)-positive cases, which account for more than 70% of all breast cancers [61]. However, ER-positive patients with metastatic disease respond poorly to tamoxifen therapy, and often with increasing dose and time develop resistance to tamoxifen [62]. In an effort to better understand the cause of tamoxifen resistance in breast cancer therapy, Xue et al. (2016), found that *HOTAIR* accumlated in nuclei and its expression was increased in tamoxifen-resistant breast tumor cells compared to primary, hormone-naïve tumor cells [15]. When the estrogen receptor alpha positive (ER+) human breast cancer cell lines (MCF7 and T47D) were treated with tamoxifen, *HOTAIR* levels increased over that of untreated cells [12]. This increase in *HOTAIR* levels correlated with a reduction in the expression of the growth regulation by estrogen in breast cancer 1 (GREB1) gene, a known ER-induced gene. Moreover, the authors observed a direct interaction of *HOTAIR* with nuclear hormone receptor ER, even under estrogen-depleted conditions. In the presence of estrogen, it was found that *HOTAIR* expression decreased in a time-dependent manner in both MCF and T47D cells, while GREB1 expression increased. Furthermore, the levels of *HOTAIR* were considerably restored in breast cancer cells following hormone deprivation, whereas GREB1 expression was lost [12]. To determine whether estrogen inhibited *HOTAIR* expression through direct ER binding to *HOTAIR* regulatory elements, the authors performed chromosome conformation capture experiments and found estrogen-induced DNA looping between the transcription start site of the *HOTAIR* gene and the ER-bound enhancer, which leads to repression of *HOTAIR* expression. *HOTAIR* possess an ER-binding site in a genomic region ∼14.5 kB upstream of the transcriptional start site of the *HOTAIR* gene [63], which is typically occupied by H3K4me1 and H3K27ac modified histones, which normally upregulate gene expression. Thus, these results suggest that *HOTAIR* is repressed by estrogen and is therefore up-regulated following hormone deprivation and in tamoxifen-resistant breast cancer, as tamoxifen is known to compete with estrogen to inhibit estrogen-induced ER activities in mammary cells [61, 64].

Importantly, over-expression of *HOTAIR* resulted in increased ER protein levels in the nucleus even under hormone-starved conditions [12]. Thus, ER-induced genes were significantly up-regulated following *HOTAIR* over-expression, even in the absence of estrogen. When up-regulated, *HOTAIR* interacts with the Polycomb group protein, EZH2, a component of PRC2 (Figure 2). The binding affinity of PRC2 with *HOTAIR* is enhanced by phosphorylation of EZH2 at Thr350 (in humans) in the G2/M phase of the cell cycle by cyclin-dependent kinase 1 (CDK1) [65]. It is thought that EZH2 allows transcription of certain genes that are targets of estrogen, which prevents direct binding of estrogen to the regulatory elements of *HOTAIR* (Figure 2). Consistent with the roles of *HOTAIR* described above, depletion of *HOTAIR* was found to inhibit cell growth and colony-forming potential in tamoxifen-resistant breast cancer cells following exposure to tamoxifen [12]. Thus, these results suggest that *HOTAIR* is involved in conferring tamoxifen resistance to breast cancer cells by promoting the transcriptional activation of the ER, in a ligand-independent manner, providing a potential therapeutic target.

### Colon cancer associated transcript 2 (CCAT2)

*CCAT2* is an intergenic lncRNA mapping to chromosome 8q24.21, and has been implicated in a variety of cancers. This lncRNA has been reported to be up-regulated in cervical squamous cell carcinoma [66], hepatocellular carcinoma [67], gastric [68], prostate [69], ovarian [70] and gallbladder cancer [71]. Moreover, it has been found to promote tumorigenesis, invasion, metastasis, and chromosomal instability in colorectal cancer [72]. *CCAT2* is also thought to be involved in the development of tamoxifen resistance in breast cancer [16] (Figure 1). Similarly, its expression was identified to be up-regulated (∼6.5-fold) in tamoxifen-resistant *versus* tamoxifen-sensitive MCF-7 and T47D breast cancer cell lines; and as expected, knockdown of *CCAT2* facilitated apoptosis and necrosis in tamoxifen-resistant cells [16]. It has been reported that the ERK/MAPK signaling pathway plays an important role in tamoxifen resistance in ER-positive breast cancer cells [13] (Figure 2). In the presence of a specific inhibitor of the ERK/MAPK pathway (U0126), the expression of *CCAT2* decreased significantly in the tamoxifen-resistant breast cancer cells compared to normal cells, which suggest that the ERK/MAPK pathway regulates the expression of *CCAT2* (Figure 2). Thus, these findings suggest that *CCAT2* is involved in tamoxifen resistance in breast cancer cells and may provide a novel therapeutic molecule in the treatment of breast cancer and/or tamoxifin-resistant breast cancer patients. However, further study is warranted in breast cancer patients to consider *CCAT2* as a useful biomarker.

### Breast cancer anti-estrogen resistance 4 (BCAR4)

*BCAR4* is a relatively newly discovered lncRNA located on chromosome 16p13.13. As the name suggests, it was first identified in breast cancer [73], where its role was analyzed in association with tumorigenesis and chemoresistance to tamoxifen [18] (Figure 1). Recently, it was reported that *BCAR4* expression negatively correlates with breast cancer disease progression. To investigate the role of *BCAR4* in tamoxifen resistance in breast cancer, Godinho et al. (2010) analyzed the expression patterns of *BCAR4* in 280 ERα-positive breast cancer patients with advanced disease, who were administered tamoxifen monotherapy as a first line treatment [18]. The authors found that out of 280 breast cancer patients, 81 patients (29%) showed high expression levels of the *BCAR4* gene. However, no correlation between *BCAR4* gene levels and age, menopausal status, tumor size, modal status, or adjuvant systemic treatment of the patients was observed [67]. Further, it was found that patients with high levels of *BCAR4* were at increased risk of early recurrence of diseases and suffered low survival rates compared to patients having low levels of *BCAR4*, reflecting the role of *BCAR4* in tumor aggressiveness. Previously, this same group reported that over-expression of *BCAR4* gives rise to aggressive phenotypes and anchorage-independent growth in breast cancer cell lines [73]. Mechanistically, *BCAR4* can increase the phosphorylation of v-erb-b2 erythroblastic leukaemia viral oncogene homolog (ERBB)2, and ERBB3, resulting in the activation of essential downstream mediators of ERBB2/ERBB3 signaling, such as AKT and extracellular signal-regulated kinase ½ (ERK ½) [18] (Figure 2). Gene amplification and over-expression of human *erythroblastosis oncogene B* (ERBB) up-regulates the HER2 receptor on the surface of breast cancer cells, which further induces the activation of the ER co-activator, amplified in breast cancer 1 (AIB-1)/steroid receptor coactivator-3 (SRC-3), which can reduce the efficacy of tamoxifen therapy [74].

The suppression of ERBB2 by siRNA in tamoxifen-resistant breast cancer cells resulted in a decrease in the proliferative ability of the cells, which reconfirms the involvement of the ERBB2/ERBB3 pathways in the development of chemoresistance to breast cancer cells. Previously, it was shown that the paired box 2 gene product (PAX2), a transcription factor that physically associates with an ER binding site [75] within the intron of the *ERBB2* gene and inhibits the transcription of ERBB2, enhanced the sensitivity of breast cancer cells to tamoxifen treatment [18]. Breast cancer cells are also characterized by increased levels of the ER co-activator amplified in breast cancer-1 (AIB-1) oncogene, which was found to be essential for ERBB2-driven oncogenesis in mice [76, 77]. PAX2 and AIB-1 compete for the ER binding site on ERBB2 in order to regulate its transcription. The up-regulation of *BCAR4* promotes AIB-1 activation, which competitively binds and inhibits the binding of PAX2 to ERBB2 sites, and thus promotes cell proliferation, tumorigenesis, and tamoxifen resistance (Figure 2) [74]. Taken together, these data suggest that *BCAR4* plays an important role in breast tumor aggression and tamoxifen resistance, driven by the ERBB2/ERBB3 signalling pathways (Figure 2).

### Urothelial carcinoma associated-1 (UCA1)

LncRNA *UCA1* is an intergenic long non-protein coding RNA, initially identified in bladder cancer [78]. It contains three exons, and encodes for two isoforms of 1.4 kb and 2.2 kb located on chromosome 19q13.12 in humans [79]. Several studies have identified *UCA1* as an oncogenic factor in several different cancers, including breast cancer [80]. In breast cancer the 1.4 kb isoform of *UCA1* is up-regulated (20-fold) and exerts its oncogenic effect, in part by the formation of a *UCA1*-hnRNP I (heterogeneous nuclear ribonucleoprotein I) complex, which results in suppression of p27 expression and promotes cancer cell proliferation [79]. *UCA1* plays an important role in establishing chemoresistance in lung (gefitinib) [81], gastric (adriamycin) [82], breast (tamoxifen) [19], esophagus (cisplatin) [60], and ovarian (cisplatin) [83] cancers (Figure 1). Based on several studies, it is thought that *UCA1* either manipulates the mTOR signaling pathway and/or exploits an miR-18a-HIF1α feedback loop as well as the Wnt/β-catenin signaling pathway to confer tamoxifen resistance in breast cancer [19, 79].

Wu et al. found substantially higher expression levels (∼20 fold) of *UCA1* in the tamoxifen-resistant LLC2 and LLC9 breast cancer cells compared to MCF-7 cells [19]. Further, depletion of *UCA1* was shown to improve the sensitivity of breast cancer cells to tamoxifen [19], consistent with its role in tamoxifen resistance. Mechanistically, *UCA1* is thought to promote tamoxifen resistance in breast cancer cells by activating the AKT/mTOR signaling pathway, where several additional miRNAs are thought to be involved in the chemoresistance phenomenon [84]. Inhibition of some of these miRNAs (*e.g.* miR-21) promotes autophagic cell death and apoptosis by targeting PTEN via inhibition of the PI3K-AKT-mTOR axis, thereby enhancing tamoxifen sensitivity in breast cancer cells [84]. Another microRNA, miR-451a, also contribute to tamoxifen sensitivity by de-regulating the activation of p-AKT and mTOR [85] (Figure 2).

Tamoxifen can up-regulate hypoxia inducible factor alpha (HIF1α), which binds to the hypoxia response elements in the *UCA1* promoter to enhance its expression [86]. It has been reported that miR-18a, a tumor suppressor, acts as an inhibitor of HIF1α by directly targeting its 3′UTR. miR-18a can disrupt the cell cycle and DNA damage responses by regulating multiple cell-cycle associated proteins such as the ATM kinase [87]. It also inhibits Cdc42, a modulator of cellular proliferation, survival and metastasis in various cancers [88]. Over-expression of *UCA1* acts as a molecular sponge and down-regulates miR-18a by associating with the Ago2-containing RNA-induced silencing complex (RISC) [21], supporting the idea that ER-positive breast cancer cells can acquire tamoxifen resistance via a *UCA1*-miR-18a-HIFα feedback loop (Figure 2) [20]. However, the proposed mechanism is still not clear. Liu et al. [21] demonstrated an interaction between *UCA1* and the Wnt/β-catenin signaling pathway in the context of tamoxifen resistance (Figure 2). When breast cancer cells are exposed to tamoxifen, the expression of HIF1-α increases, which stimulates over-expression of *UCA1*, and thereby enhances β-catenin translocation into the nucleus, promoting the extracellular redistribution of the ER [21]. β-catenin, an intracellular transducer of the wnt/β-catenin signaling pathway, remains highly unstable and inactivated in the cytoplasm where it is subjected to phosphorylation/ubiquitination-associated proteasomal degradation [89]. Wnt/β-catenin signaling inhibits the degradation of β-catenin and allows its accumulation in the nucleus, where it binds with the T cell factor/lymphoid enhancer factor (TCF/LEF) family of transcription factors and positively regulates wnt-associated genes (Figure 2) [89]. Therefore, *UCA1*-associated ER and Wnt/β-catenin signaling contribute to breast cancer progression via tamoxifen resistance (Figure 2). Further studies revealed that the Wnt/β-catenin pathway plays an essential role in maintaining the stem-cell like characteristics of breast cancer cells contributing to metastasis and reducing the efficacy of tamoxifen therapy [21].

Together, these findings reveal that lncRNA *UCA1* induces chemoresistance to tamoxifen through different molecular pathways, and regulates several other miRNAs involved in breast cancer (Figure 2). Therefore, *UCA1* may serve as a therapeutic target for developing a potential treatment against breast cancer, particularly in tamoxifen-resistant patients.

### LncRNA-regulator of reprogramming (LncRNA-ROR)

*LncRNA-ROR* is a long intergenic non-coding RNA, with a length of 2.6 kb, comprised of four exons located on chromosome 18q21.31 [90, 91]. In humans, the *ROR* gene is regulated by certain pluripotency-inducing transcription factors such as Sox2, Nanog and Oct4, resulting in an increased expression in embryonic and induced pluripotent stem cells [91]. *LncRNA-ROR* plays an important role in regulating breast carcinoma by inhibiting the activation of different cellular pathways such as the stress-induced p53 pathway [91]. It has also been reported to be up-regulated by up to 5-fold in various cancers such as gallbladder cancer [92], gastric cancer [93], and nasopharyngeal carcinoma [90]. *LncRNA-ROR* promotes chemoresistance in head and neck [90] and breast cancers [22] against a variety of chemotherapeutic agents, including cisplatin and tamoxifen, respectively (Figure 1).

Recently, Zhang et al. (2017) analyzed the expression pattern of *lncRNA-ROR* in breast cancer cell lines using qRT-PCR, and found it to be significantly up-regulated (∼7-fold) compared to breast epithelial MCF10A cell lines [22]. To determine the effects of *lncRNA-ROR* on breast cancer cells in relation to tamoxifen resistance, the proliferation rate of tamoxifen-resistant MCF7/TR5 breast cancer was compared with MCF-7 cells. It was found that the MCF/TR5 cells showed significantly higher proliferation rates compared to the MCF-7 cells following treatment with tamoxifen. When siRNA was used to deplete ROR in naturally tamoxifen-resistance (MDA-MB-231) and tamoxifen-induced breast cancer cells (MCF7/TR5), this resulted in lower rates of proliferation following treatment with tamoxifen, again implicating *ROR* in the resistance phenotype. It was also demonstrated that cells having relatively low *ROR* expression showed more sensitivity to tamoxifen compared to cells having higher *ROR* expression. Moreover, western blot analysis revealed the contribution of *lncRNA-ROR* in breast cancer cell invasion and the EMT process, as cell lines transfected with si-lncRNA-ROR showed an increased expression of E-cadherin (an epithelial marker) and decreased expression of Vimentin (a mesenchymal marker) [22] (Figure 2), which influenced the tamoxifen resistance ability of breast cancer cells.

At the molecular level, it was found that *lncRNA-ROR* regulates the expression of ZEB1 and ZEB2 transcription factors, and as expected, the expression of ZEB1 and ZEB2 were significantly downregulated in ROR-siRNA treated MDA-MB-231 and MCF/TR5 cells [22]. As mentioned earlier, ZEB1 and ZEB2 are well known oncogenic transcription factors that play crucial roles in regulating the EMT process [94]. Principally, *lncRNA-ROR* interacts with miR-205-5p ribonucelopotein complex and functions as a molecular sponge for miR-205-5p. For example, it was reported that miR-205-5p acts as a negative regulator of the EMT process by silencing the expression of the *ZEB1/2* genes by binding to their 3′UTRs in gastric cancer [95]. To better understand its role in the context of breast cancer resistance, the authors found that upregulation of *lncRNA-ROR* in tamoxifen resistant MCF7/TR5 and MDA-MB-231 breast cancer cells inhibited the expression of miR-205-5p, and increased ZEB1/2 gene expression, thereby enhancing the EMT process (Figure 2).

Collectively, these results suggest that *LncRNA-ROR* acts as an oncogenic molecule in breast cancer, in part by inhibiting miR-205-5p and promoting the EMT process by enhancing the expression of ZEB1 and ZEB2 transcription factors, contributing to breast cancer cell invasion as well as tamoxifen resistance (Figure 2). Thus, *LncRNA-ROR* may potentially serve as a biomarker for assessing the sensitivity of breast cancer cells to tamoxifen, though further research is required to explore it's role *in vivo*.

### H19

*H19*, an imprinted maternally expressed transcript (∼2.3 kb), is located on chromosome 11p15.5. It is classified as a non-protein coding RNA that plays an essential role in mammalian development [96]. Several investigations have confirmed that *H19* is over-expressed in bladder [97], glioma [98], thyroid [99], gastric [100] and breast cancers [101] through various mechanisms, including chromosomal abnormalities, transcription factor binding, and epigenetic alterations. High levels of *H19* often correlate with poor prognosis for cancer patients, and with the development of chemoresistance in various cancers, including ovarian (cisplatin) [102], colorectal (methotrexate) [103], liver (doxorubicin) [104] lung (cisplatin) [105], and breast (paclitaxel) [17] (Figure 1).

The expression of *H19* was found to be highly up-regulated in paclitaxel induced ERα-positive resistant breast cancer cell lines MCF-7R and ZR-75-1 (by ∼9-fold and 4-fold, respectively) compared to their parental lines MCF-7S and ZR-75-1S [17]. While no up-regulation was observed in the ERα-negative breast cancer cell line MDA-MB-231 even in the presence of paclitaxel [17]. Similarly Lu et al. [106] demonstrated a higher expression (3-fold) of *H19* in ERα-positive breast cancer patients (n = 76) than in ERα-negative breast cancer patients (n = 53), which suggests a possible involvement of ERα in the up-regulation of *H19* in chemoresistant cancer cells. Since ERα is considered a powerful chemoresistance factor, the authors examined whether depletion of *H19* could attenuate ERα-induced drug resistance. They found that suppression of *H19* expression sensitized MCF-7S cells to paclitaxel, suggesting that ERα promoted *H19* expression in breast cancer cells and that *H19* is an important mediator of ERα-induced drug resistance [17]. The contribution of *H19* was further verified in MCF-7R and ZR-75-1R cells transfected with *H19*-targeting siRNA and treated with different concentration of paclitaxel for 48 h. By MTT assays, it was observed that both MCF-7R and ZR-75-1R cells showed decreased viability following treatment with paclitaxel, suggesting that *H19* contributes to drug resistance in breast cancer. Previous studies reported that *H19* induces P-glycoprotein expression under hypoxic conditions, which promotes the cellular efflux of specific drugs, including paclitaxel and epirubicin [101]. It was observed that knockdown of *H19* significantly reversed resistance even to drugs that were not substrates of P-glycoprotein, indicating *H19* induced breast cancer chemoresistance through multiple mechanisms. It was also found that *H19* attenuated apoptosis by inhibiting the transcription of the pro-apoptotic proteins BCL-2 interacting killer (BIK) and the BCL-2 homology domain 3 (BH3) protein, NOXA [107]. The protein levels of BIK and NOXA increased in *H19*-depleted cells, which increased the apoptotic ratio of the cells [17]. Furthermore, the over-expression of BIK or NOXA (by 2.5-fold) in MCF-7R cells decreased the survival of paclitaxel-resistant cells via increased apoptosis [17]. The authors also demonstrated that *H19*, by recruiting EZH2, an essential component of PRC2, catalyses the trimethylation of H3K27 in the promoter region of BIK, suppressing its transcription (Figure 2). However, how *H19* inhibits the transcription of NOXA is not yet clear.

Taken together, these results suggest that the ERα-H19-BIK axis plays an important role in contributing to drug resistance in breast cancer chemotherapy. Thus, modulation of the components of this signalling axis may be used to overcome paclitaxel resistance in breast cancer patients.

### LncRNA-adriamycin resistance associated (LncRNA-ARA)

*LncRNA-ARA* is a 4,957 nt unspliced, polyadenylated transcript transcribed from the intron of the p21-activated kinase 3 *(PAK3)* gene at chromosome Xq23 [23]. Jiang et al. through genome-wide lncRNA microarray analysis, reported the differential expression of *lncRNA-ARA* in adriamycin resistant MCF-7/ADR breast cancer cells compared to parental MCF-7 cells [23]. The authors have shown that depletion of lncRNA-ARA by ARA-siRNA sensitized MCF-7/ADR cells to adriamycin, which suggests that lncRNA-ARA may be a candidate molecule in the development of resistance against adriamycin in breast cancer cells. The authors also demonstrated that the endogenous expression of *lncRNA-ARA* was upregulated by ∼13-fold at 48 hours following treatment with adriamycin in sensitive MCF-7 cells, whereas no significant change was observed at same time point and drug concentration in MCF-7/ADR cells, indicating that drug treatment might initially induce ARA expression during the development of drug resistance. It was further observed that siRNA-mediated silencing of *lncRNA-ARA* inhibited cellular proliferation, induced a G2/M arrest, and enhanced cell death via apoptosis, necrosis, and migration defects in adriamycin-resistant cells. Also, ARA depletion resulted in the upregulation of the pro-apoptotic Bcl-2 Associated X (BAX) protein and the downregulation of the anti-apoptotic B-cell lymphoma extra-large (Bcl-xL) protein, hence playing a role in apoptosis and cell death. By using bioinformatics tools such as gene ontology (GO) and pathway mapping tools such as Kyoto encyclopaedia of genes and genomes (KEGG), lncRNA-ARA was found to regulate multiple oncogenic signalling pathways such as purine metabolism, pyrimidine metabolism, focal adhesion, cell cycle, PPAR signaling and MAPK signaling in cells, all of which have been reported to contribute to acquisition of adriamycin resistance in breast cancer [4, 108–110].

Taken together, the above findings suggest a novel role of *lncRNA-ARA in* contributing to adriamycin resistance in breast cancer cells, although further study is required, especially in breast cancer patients.

## CONCLUSIONS

It is very disheartening that even with the availability of many chemotherapeutic regimens for the treatment of breast cancer (Figure 1), the main causes of breast cancer-related deaths include recurrence and metastases [2]. Accurately predicting those patients at high risk for recurrence may afford improved monitoring and treatment for breast cancer patients. The dysregulated expression and stabilization of lncRNAs (Table 1) has recently been established as a critical factor for modulating epigenetic changes in immune signaling pathways, DNA repair pathways, cell death pathways and energy metabolism, particularly in rendering tumor cells refractory to chemotherapeutic interventions. Here, we discussed how lncRNA pools can influence the expression of various oncogenes and cancer signaling pathways, and can also affect the angiogenesis, metastasis, immune evasion, and EMT processes involved in cancer etiology and progression (Figure 2).

In conclusion, we are hopeful that this review will contribute to the better understanding of the development of chemoresistance in breast cancer cells and the roles of long non-coding RNAs in these processes. Thus, with further work in this field, lncRNAs may be developed as a biomarkers for the detection and/or prevention of breast cancer. Additionally, lncRNA-based approaches may provide an additional treatment modality in personalized medicine alone or in combination with existing tumor directed interventions to improve patient outcome. Thus, lncRNA molecules may represent a “next generation” therapy option for breast cancer patients.
